# Oxidative discolouration in whole-head and cut lettuce: biochemical and environmental influences on a complex phenotype and potential breeding strategies to improve shelf-life

**DOI:** 10.1007/s10681-017-1964-7

**Published:** 2017-07-18

**Authors:** Paul J. Hunter, Laura D. Atkinson, Laura Vickers, Stella Lignou, Maria Jose Oruna-Concha, David Pink, Paul Hand, Guy Barker, Carol Wagstaff, James M. Monaghan

**Affiliations:** 1 Harper Adams University, Newport, Shropshire TF10 8NB UK; 2 Monsanto UK Ltd, Cambridge, CB23 6DW UK; 3 Department of Food and Nutritional Sciences, University of Reading, Whiteknights, PO Box 226, Reading, Berkshire RG6 6AH UK; 4 School of Life Sciences, University of Warwick, Gibbet Hill Road, Coventry, CV4 7AL UK

**Keywords:** Lettuce, Discolouration, Biochemistry, Environment, Polyphenols, Phenylpropanoid pathway, Review

## Abstract

Lettuce discolouration is a key post-harvest trait. The major enzyme controlling oxidative discolouration has long been considered to be polyphenol oxidase (PPO) however, levels of PPO and subsequent development of discolouration symptoms have not always correlated. The predominance of a latent state of the enzyme in plant tissues combined with substrate activation and contemporaneous suicide inactivation mechanisms are considered as potential explanations for this phenomenon. Leaf tissue physical properties have been associated with subsequent discolouration and these may be influenced by variation in nutrient availability, especially excess nitrogen and head maturity at harvest. Mild calcium and irrigation stress has also been associated with a reduction in subsequent discolouration, although excess irrigation has been linked to increased discolouration potentially through leaf physical properties. These environmental factors, including high temperature and UV light intensities, often have impacts on levels of phenolic compounds linking the environmental responses to the biochemistry of the PPO pathway. Breeding strategies targeting the PAL and PPO pathway biochemistry and environmental response genes are discussed as a more cost-effective method of mitigating oxidative discolouration then either modified atmosphere packaging or post-harvest treatments, although current understanding of the biochemistry means that such programs are likely to be limited in nature and it is likely that they will need to be deployed alongside other methods for the foreseeable future.

## The value of lettuce

The UK processed salad sector was valued at approximately £848 million in 2009 (Soininen 2009), with underpinning UK lettuce (*Lactuca sativa*) production worth approximately £155 million and additional lettuce imports worth in excess of an additional £160 million in 2013 (Defra 2014). Much of this lettuce is purchased in ‘ready-to-eat’ pre-prepared salad packs of either lettuce or mixed salad containing lettuce (Soliva-Fortuny and Martín-Belloso 2003). Cut salads however, are highly perishable; delivering an increasing supply of high quality salad product poses considerable challenges and significant wastage can occur from producer through to retailer and consumer (amounting to approximately £270m: WRAP 2012). Consequently, breeders and producers are attempting to reduce wastage (thus increasing marketable yield) by improving post-processing quality of packaged products.

The occurrence of discolouration in fresh leafy vegetables has been reported as a severe problem for the food industry as a whole (Chiesa 2003). Losses due specifically to cut-surface discolouration are hard to quantify although anecdotal evidence suggests that this can account for substantial customer complaints at certain times of the year and rejections of whole batches of heads, particularly with imported crops, where products may take longer to reach the consumer from harvest. Processing operations (such as cleaning/washing of heads, trimming, coring, cutting, and drying by centrifugation) can cause mechanical damage to leaves and may facilitate this discolouration (Kays 1999; Hilton et al. 2009). In addition, there are numerous pre-harvest factors, including plant physiology, agronomic practices and environmental variation that can contribute to product discolouration (particularly the accumulation of red or pink pigments at sites of tissue damage or cut surfaces), although symptoms may not develop until plants are either in storage as whole heads or the cut surfaces of processed lettuce are exposed to oxygen. The use of modified atmosphere packaging (MAP) can delay the development of cut-surface discolouration thereby extending product shelf life (Brecht et al. 2003; Singh 2010). This is an added expense during processing and once MAP is opened, exposure to oxygen can lead to the rapid onset of discolouration (Tudela et al. 2016), meaning product shelf life is reduced from the consumer perspective.

Whilst processing techniques have a role to play in reducing additional damage to the cut leaves, genetic variation is also a major factor in determining post-harvest quality of ready-to-eat lettuce. This review first summarises the biochemistry of cut-surface discolouration, then considers the effect of the environment, both seasonal and agronomic on these processes, and hence phenotype, followed by a summary of the current understanding of the genetic control of discolouration. Finally, the role of breeding strategies to develop cultivars with either reduced discolouration potential or delayed onset of discolouration phenotype is explored.

## Biochemistry of oxidative discolouration

Cut surface discolouration in leafy vegetables is widely attributed to the oxidation of phenolic compounds to produce quinones, which are then further oxidized to produce coloured compounds (Martinez and Whitaker 1995; Payne et al. 2006). The production of quinones has been shown to be enzymatic in nature, whilst the secondary oxidation is largely non-enzymatic. Quinones are highly reactive molecules that can polymerize, or react with amino acids and proteins to form brown, red, or even black pigments (Joslin and Pointing 1951; Zawistowski et al. 1991; Martinez and Whitaker 1995; Solomon et al. 1996; Karpeta 2001; Dincer et al. 2002; Aydemir 2004; Fan et al. 2005; Gawlik-Diziki et al. 2008; Toivonen and Brummell 2008). Whilst the accumulation of these pigmented compounds is often referred to as browning, the most significant colour present in lettuce after seven days of storage is often red or pink (referred to as pinking) (Cantos et al. 2001; Lewis 2001).

The enzymatic phase of this process is generally considered to be driven by the action of polyphenol oxidase (PPO), also known as tyrosinase. The activity of this enzyme in plant tissues does not, however, always correlate with the subsequent development of discolouration in lettuce (Ke and Saltveit 1989; Couture et al. 1993; Heimdal et al. 1995; Castañer et al. 1999; Cantos et al. 2001; Mai and Glomb 2013). It is possible that discrepancies between different studies are a reflection of a paradigm which is an over-simplification of the processes involved.

The interaction of PPO with phenolic compounds is the culmination of two complex biochemical processes which do not interact under normal plant growth conditions due to separate compartmentalization within the plant tissues. Physical damage can disrupt the sub-cellular compartmentalization at the wound surface. This then results in release and intermixing of PPO and phenolic substrates (Degl’Innocenti et al. 2005; Querioz et al. 2008; Toivonen and Brummell 2008) and subsequent discolouration (Toivonen 2004).

Phenolic compounds are synthesized via the phenylpropanoid pathway. This is a secondary metabolic pathway producing non-essential metabolites. The initial and rate-determining step is the conversion of phenylalanine to *trans*-cinnamic acid by the enzyme phenylalanine ammonia-lyase (PAL) (Wanner et al. 1995). There are subsequent specific branch pathways for the formation of all major classes of phenylpropanoid compounds including monolignols/lignin, sinapate esters, condensed tannins, anthocyanins, coumarins, benzoic acids, flavonoids/isoflavonoids and stilbenes (Dixon et al. 2002). Total phenolic content in damaged lettuce has been shown to increase over 24 h, even when subsequently stored at 4 °C (Cantos et al. 2002) and the concentrations of specific phenolic compounds as well as the activity of PAL have been shown to increase within 4 h of wounding in iceberg lettuce (Ke and Saltveit 1989). The enzymes involved in these processes tend to be either incorporated into the membrane of, or loosely associated with, the endoplasmic reticulum (ER). Once formed, phenolic compounds are transported from the ER in membrane vesicles (Toivonen and Brummell 2008) and primarily directed to either the vacuole or the cell wall, although phenolic compounds may be found at low levels in cytoplasm, apoplast and the mitochondria; these latter compounds are, however, normally associated with specialised functions.

In plant tissue, PAL is typically induced in response to wounding, increasing downstream biosynthesis of polyphenols (Hyodo et al. 1978; López-Galvez et al. 1996; Peiser et al. 1998; Hisaminato et al. 2001) Such increases have been identified in several lettuce cultivars with chlorogenic (5-caffeoylquinic) acid being the major polyphenolic compound produced, although increases in levels of chicoric (dicaffeoyltartaric) and isochlorogenic (3,5-dicaffeoylquinic) acids were also identified (Tomás-Barberán et al. 1997; Cantos et al. 2002). Increases in PAL activity after wounding have been found to be closely related to the development of russet spotting in whole head lettuce (Hyodo et al. 1978; Ke and Saltveit 1989), however, despite the observed increases in the levels of polyphenolic compounds, no correlation with discolouration was observed in processed lettuce (Castañer et al. 1999; Cantos et al. 2001). This is supported by the observation that following lettuce wounding, the total activity of the other key enzyme in the process, PPO, remained constant, suggesting no de novo PPO synthesis in response to physical damage (Chazarra et al. 1996).

PPO is a copper-requiring metallo-enzyme associated with (although not found to be intrinsic to) the thylakoid membranes of the chloroplast in many plants including lettuce (Sommer et al. 1994; Chazarra et al. 1996; Wahler et al. 2009). PPO catalyses two distinct reactions; the *o*-hydroxylation of monophenols to produce *o*-diphenols (monophenolase or cresolase activity) and the subsequent oxidation of the *o*-diphenols to *o*-quinones (diphenolase or catecholase activity), both of which require the presence of molecular oxygen (López-Galvez et al. 1996; Chazarra et al. 2001; Toivonen and Brummell 2008). The initial monophenolase reaction is slow (therefore rate-limiting) and results in colourless products, while the diphenolase reaction is rapid and results in quinones and subsequently, coloured compounds (Toivonen and Brummell 2008).

PPO from a wide range of fungal and plant sources (including lettuce), show evidence of both active and latent states (Mayer and Harel 1979; Chazarra et al. 1996; van Gelder et al. 1997; Cabanes et al. 2007). Chazarra et al. (1999) showed that monophenolase activity in lettuce PPO follows the model developed by Cabanes et al. (1987) for PPO from other sources. In brief, PPO exists in three forms depending on the oxidation state of the copper-containing catalytic centre. Two of these forms (the *met* and *oxy* forms) are particularly important in the monophenolase activity. In the *oxy* form, *o*-quinones are produced from monophenols; the *met* form however has no monophenolase activity and the binding of monophenols to this form results in the removal of PPO from the active cycle. Such inactivated PPO can be reactivated by the action of *o*-diphenols. *o*-diphenols are the intermediate state between monophenol and *o*-quinone, but some *o*-quinone products are also recycled chemically to *o*-diphenols. Consequently, the observed latent state may correspond to the *met* form of the enzyme. The lag phase observed in PPO enzyme kinetics can be attributed to the time required for the rate of *o*-diphenol production via recycling to reach that of *o*-diphenol metabolism to *o*-quinone. The existence and duration of this lag phase may in part explain the observed lack of correlation between PPO activity and discolouration: the PPO is predominantly in the latent form when measured (Winters et al. 2008; Cabanes et al. 2007), and requires activation in vivo in order to subsequently produce discolouration. Difficulties assaying the monophenolase activity due to latency may also explain the suggestion in the literature that plant PPOs could be divided into “catecholases”, which only possess the diphenolase activity and “tyrosinases”, which show the monophenolase activity in addition (Boeckx et al. 2015).

A number of factors that can switch the enzyme from latent to active state have been identified; these include pH change, proteolytic cleavage and the detergent sodium dodecyl sulphate (SDS). Chazarra et al. (1999) suggested that pH may influence the enzyme affinity for the substrate. They observed that altering reaction conditions from pH 3.6 to 5 resulted in an increase in monophenolase activity and a reduced lag phase. This was attributed to a decreasing affinity of the monophenolase-inactive form of PPO for monophenol ligands (i.e. increasing the dissociation rate resulting in less PPO being removed from the cycle) and/or increasing the affinity of the monophenolase-active form of PPO for the same substrate, thus increasing turnover.

In relation to proteolysis, PPO from a number of sources has been shown to be activated in vitro by trypsin cleavage. Flurkey and Inlow (2008) identified an N-terminal and a C-terminal processing site in the PPO protein. They suggested that cleavage at these sites exposed an N-terminal tyrosine residue and a C-terminal arginine residue which interact to stabilize the protein secondary structure. They also noted that the N-terminal cleavage site was consistent with removal of the N-terminal transit peptide for thylakoid associated proteins. Furthermore, Virador et al. (2010) noted that N-terminal processing of PPO in grape was associated with release of the PPO from the thylakoid into the chloroplast stroma, however both active and latent forms of PPO have been isolated from membrane-bound and soluble fractions of peach extract (Cabanes et al. 2007) suggesting that N-terminal processing is not directly responsible for PPO activation. Sellés-Marchart et al. (2007) noted that trypsin activation of PPO from loquat fruit altered the pH range of the enzyme. A set of protein domain-swap experiments by Leufken et al. (2015) have shown that this response may be associated with the C-terminal region of the protein suggesting that this domain (attached to the N-terminal region by a flexible linker domain) may physically block access to the active site of the enzyme under certain pH conditions (Boeckx et al. 2015; Molitor et al. 2016).

There is evidence that the presence of SDS, may shift the pH optimum of PPO toward more neutral pHs (Chazarra et al. 1997; Dirks-Hofmeister et al. 2012), resulting in an activation of PPO at these pHs and an inactivation of the enzyme under more acid conditions (Jiménez and García-Carmona 1995). Whilst SDS is not a natural component of plant cells, it has been suggested that plant lipids may activate the latent PPO activity *in planta* in a similar manner (Hutcheson and Buchanan 1980; Golbeck and Cammarata 1981; van Gelder et al. 1997). In addition, Gandia-Herrero et al. (2005) noted that trypsin-activated PPO was no longer susceptible to activation by SDS. The experiments by Leufken et al. (2015) identified the linker domain between the proteolytically cleaved C-terminus and the N-terminal region of the protein as being associated with SDS sensitivity. Since the linker domain is not cleaved during proteolysis it should theoretically still be available for interaction with SDS however, many of the of these activation mechanisms appear to be associated with a conformational change in the PPO protein (Chazarra et al. 2001; Sellés-Marchart et al. 2007) and it may be that the C-terminal cleavage results in such a change meaning that either the SDS binding site on the linker domain is no longer physically available for interaction with SDS or that the configuration is now such that SDS binding no longer results in exposure of the enzyme active site.

In lettuce, Chazarra et al. (2001) have shown that activation can occur slowly in the presence of the enzyme’s substrate (in this case chlorogenic acid) and similar substrate activations have been demonstrated for a range of monophenols in PPO from red clover (Winters et al. 2008). This has been suggested as the more likely in vivo activation mechanism for latent PPO, although proteolytic activation in vivo has also been demonstrated for red clover (Winters et al. 2008). In addition to substrate activation, PPO from various sources, including lettuce has been shown to suffer from suicide inhibition, i.e. irreversible inhibition by a substrate analogue that occurs during the natural metabolic process (Chazarra et al. 1999; Land et al. 2007). In tyrosinase from *Agaricus bisporus*, these have been identified as catechols (*o*-diphenols) of various specific structures (Land et al. 2007). Oxidation of diquinones produced at cut surfaces could theoretically lead to the production of small amounts of inhibitory catechols.

The development of discolouration in cut lettuce becomes even more complicated when the fact that PPO has different affinities for and reaction rates with different substrates (Złotek and Gawlik-Dziki 2015) is taken into account. Since it has been noted that the pigment resulting from PPO oxidation of polyphenolics is also dependent on the initial substrate (Toivonen and Brummell 2008) it seems likely that the relative amounts of polyphenolic components downstream of PAL activity in the polyphenolic pathway may also be important in determining not only the overall activity of PPO but the final pigmentation produced and the speed of discolouration development. This suggests a possible involvement for the steps in this pathway in the distinction between pinking and browning symptoms seen in lettuce discolouration.

In a further complication, some authors have attributed a partial role in the browning response to peroxidases (POD) (Richard-Forget and Gauillard 1997; Degl’Innocenti et al. 2005). POD enzymes are part of the lignification process at wound sites and require the presence of hydrogen peroxide (H_2_O_2_). Polymerized lignin imparts a brown colour to tissues of itself, irrespective of any colour associated with oxidation of polyphenolics and since the monolignol precursors to lignin are products of the phenylpropanoid pathway, variations in the relative abundance of compounds in this pathway may impact on levels of lignin. H_2_O_2_, availability may also be subject to such impacts since one source of H_2_O_2_ has been suggested to be PPO-mediated oxidation of polyphenols (Jiang and Miles 1993; Tomás-Barberán and Espín 2001), with the structure of the polyphenol substrate affecting potential H_2_O_2_ yield (Cantos et al. 2002). Cantos et al. (2001) have reported POD induction in six lettuce cultivars following cutting and several authors have reported that treatments that reduce browning in lettuce are also associated with reduced POD activity (Mousavizadeh and Sedaghathoor 2011; Kim et al. 2014; Chen et al. 2017). There is however, no direct evidence for a causal relationship between POD and lettuce discoloration and it remains unclear whether POD-mediated browning is consistently a significant component in fresh produce discolouration.

A final layer of complexity is the possibility of “revealed discolouration” due to chlorophyll bleaching, the mechanisms of which are reviewed by Toivonen and Brummel (2008). The strong green colour of chlorophyll may mask developing pinking or browning discolouration with chlorophyll degradation during storage potentially revealing the underlying discolouration. This is likely to be more of an issue in processed lettuce than whole-head lettuce (Brown et al. 1991) as chlorophyll breakdown in cut salads has been shown to be predominantly driven by either oxidative breakdown of fatty acids via the enzyme lipoxygenase (Thomas 1986) or peroxidative breakdown of *p*-hydoxyphenolic compounds by POD (Martinoia et al. 1982; Yamauchi et al. 2004). A second, much slower and oxygen-independent pathway also removes chlorophyll by the actions of dechelatase and chlorophyllase. Whilst slower and therefore apparently less important in respect of the shelf-life constraints of cut salads, the resultant intermediate compounds of this pathway, pheophorbide and pheophytin are themselves brown coloured and may contribute to the appearance of browning (Matile et al. 1999).

## Influence of environment

Since cellular metabolism does not stop in lettuce heads at harvest but continues in the tissues throughout storage, the environment the plant encounters during growth has the potential to influence product shelf life post-harvest (Lee and Kader 2000). Pre-harvest environmental influences on post-harvest discolouration phenotype can be categorized as managed agricultural practices and unmanaged climatological and microbial factors.

### Managed agricultural practices

#### Physical damage

Lettuce is a physically fragile crop and mechanical damage either during harvest or post-harvest, frost damage sustained during growth (particularly in early or late season crops) or cellular disruption due to exceptionally rapid or abnormal growth can all affect the post-harvest storage potential of lettuce (Sharples 1965). Zhang et al. (2007) identified strong negative correlations between discolouration and the plasticity and elasticity of leaf tissue, and a positive correlation with break strength, suggesting that subsequent discolouration may be related the ability of the lettuce tissue to deform in response to physical pressure. Not only may wound sites discolour, either during crop production or in storage, but the increased phenolic levels and enzyme activities produced in response to wounding may represent increased discolouration potential if apparently healthy material, damaged shortly before or during harvest, is later cut for prepared salad production.

#### Nutrient

Nutrients have a major effect on the rate and quality of plant growth. The application of an appropriately balanced nitrogen: phosphorus: potassium (NPK) regime can significantly improve cut salad shelf life, however if excess N is applied, the shelf life of both whole head and processed lettuce can be significantly reduced (Poulsen et al. 1995; D’Antuono and Neri 2001; Rogers et al. 2006; Monaghan et al. 2010). Some studies, have reported that whole head harvest and post-harvest quality traits, including pinking, showed no significant differences under different N management strategies (Hartz and Breschini 2001; Hilton et al. 2009). These differences may however be a reflection of variations in soil properties, cropping history or irrigation regime, all of which have been shown to influence the levels of available N after fertilisation (Jackson et al. 1994; Cameron et al. 2013; Kruse and Nair 2016). N levels influence, amongst other attributes, cell size and number, as well as the proportion of cell wall material volume to total tissue volume (Steenhuizen and van der Boon 1985). The reduction in shelf life observed may be a consequence of such changes leading to less robust tissue and consequently significantly more physical damage occurring in processed leaves produced under excess N.

Although phosphorous (P) levels are generally maintained in agricultural soils to ensure good plant growth and yield, P application has also been reported as being able to improve shelf life in lettuce (Yano and Hayami 1978). Typically, P is applied as superphosphate (calcium phosphate) leading to the associated addition of calcium (Ca). The effect of Ca on shelf life and discolouration appears to be complex. Ca increases firmness in many fruits and vegetables by cross-linking pectins in the cell wall (Grant et al. 1973), indicating a role in resisting physical damage. A reduction of discolouration in processed lettuce has been associated with Ca application under low K:N ratios (Hilton et al. 2009) suggesting that the effects of Ca may be heavily dependent on other factors. Deficiency of Ca itself however, has long been associated with a browning disorder in lettuce called tip-burn or tip-scorch (Collier and Tibbitts 1982). This is believed to occur due to Ca-deficiency induced leakage of plant cell membranes under conditions of rapid growth (Saure 1998). Chutichudet et al. (2009) reported that soil amendment with CaSO_4_ had no significant effect on the levels of total phenolic compounds or quinones in lettuce tissue, however Borghesi et al. (2013) found that increasing levels of Ca applied to hydroponic nutrient solution resulted in an increase in phenolic content to an optimum of 15 mM Ca, above this concentration, phenolic levels began to reduce. The authors attributed the phenolic increase to adaptation to mild salt stress.

#### Maturity

Lettuce can accumulate 70% of the final fresh weight during the 21 days prior to harvest (Zink and Yamaguchi 1962). Storage life has been related to lettuce maturity, with immature lettuces exhibiting lower storage life (Kasmire and Cantwell 1992) and greater levels of whole head discolouration (browning) than mature heads (Barg et al. 2009). The volume and density of heads determine maturity, with discolouration in whole head lettuce being negatively correlated with both diameter and height (determinants of volume, suggesting a positive correlation with head density) (Jenni et al. 2008). In contrast, Hilton et al. (2009) showed that pinking is more prevalent in processed over-mature lettuce than in tissue from young or mature plants and Kang et al. (2008) reported that over-mature (but pre-bolting) lettuce heads showed higher levels of whole-head discolouration than mature heads, although less severely than pre-mature heads. Levels of phenolic compounds associated with PPO-mediated discolouration, have also been shown to be similar in immature, mature and over mature lettuce heads (Wurr et al. 2003). A significant distribution gradient for phenolic compounds has been shown in lettuce however, with the outer leaves accumulating more phenolic compounds than the inner leaves (Viacava et al. 2014) and being more susceptible to browning (Iakimova and Woltering 2015). Since lettuce heads continue to grow from the centre when formed, the outer leaves are the oldest, thus suggesting that phenolic content may increase with leaf maturity. As the head matures, however, the majority of the biomass will be composed of lower phenolic content inner leaves. Furthermore, the outermost leaves with the highest phenolic content are more likely to be trimmed away during processing, thus reducing the overall level of phenolic content in the head or processed material. It is possible that this trimming may be more extensive in “over-mature” heads compared to heads harvested at the optimal level of maturity and this could lead to a reduction in the observed severity of discolouration in processed lettuce where all the leaves from different depths in the head are mixed together.

#### Water

Lettuce tissue is approximately 95% water, consequently the water content of the tissue is related to saleable weight (Monaghan et al. 2016). Reducing irrigation in lettuce reduces marketable yield (Gallardo et al. 1996; Karam et al. 2002; Monaghan et al. 2016), therefore lettuce crops are typically well irrigated up until harvest in order to maximize yield (Rogers et al. 2006). Mild water-deficit stress however, has been shown to reduce cut-surface browning on mid-ribs (Luna et al. 2012) and mid-rib discolouration in whole head lettuce (Monaghan et al. 2016). The method of irrigation delivery may also be important: Trickle-irrigation has been shown to deliver an improvement in shelf life (albeit at a yield cost) compared to water delivery by overhead-spray (Rogers et al. 2006), possibly by inducing a mild water-deficit because of the smaller volumes delivered. Agüero et al. (2008) however, have shown that neither absolute water content or relative water content (relative to the total water holding capacity of the tissue) are significantly correlated with variation in overall lettuce quality (which includes a measure of discolouration). Water-deficit stress has also been shown to increase the concentration of certain phenolic compounds in lettuce, particularly chicoric acid (Oh et al. 2010) and to cause the accumulation of antioxidant compounds (Sofo et al. 2005; Oh et al. 2010), which may potentially inhibit oxidation of phenolics and thus discolouration development. Whilst there is no evidence to specifically associate increased levels of chicoric acid with a reduction in the level of lettuce discolouration, the different pathways involved in phenylpropanoid metabolism are interlinked (Treutter 2010), consequently, the diversion of plant resources into one pathway (i.e. increasing production of chicoric acid) may result in a reduction of resources available for other branches, potentially leading to reduced levels of compounds which may be more directly involved in discolouration.

In addition to water-deficit stress, excess water during the growing period (e.g. from rainfall events, which cannot be controlled) can also have a significant impact on lettuce shelf life, increasing both caffeic acid levels and PAL and PPO activity, resulting in increased browning in processed lettuce (Luna et al. 2012). Irrigation can also have a large effect on the mechanical properties of the lettuce leaf by influencing cell expansion and tissue turgor pressure (Kuslu et al. 2008). Higher relative water content in whole head tissue at harvest increases susceptibility to damage during processing and subsequent pinking development. Such susceptibility may be the result of increased turgor pressure in the cells, resulting in leaf tissue being more susceptible to physical damage (Alzamora et al. 2000; Abbot and Harker 2016).

### Unmanaged climatological and microbial factors

Although certain aspects of the plant growing environment can be manipulated by producers, there are other aspects of the environment which are beyond conventional control. In addition to excess irrigation from rainfall events discussed above, other factors not easily controlled include temperature and light.

#### Temperature

For cool season vegetables, such as lettuce, temperature variation can have a number of effects. Lower growing temperatures have been associated with reduced discolouration (Tudela et al. 2017), with pinking in both cut and whole-head lettuce (Jenni 2005) and phenolic content of baby-leaf lettuce (Marin et al. 2015) being significantly influenced by the temperature experienced by the plants just prior to harvest. Whether the day or night temperature is more important in influencing lettuce discolouration remains unclear. A positive correlation between minimum temperature (i.e. night-time minimum) one week prior to harvest and the proportion of whole-head lettuce showing discolouration has been reported, suggesting that it is night-time temperatures (i.e. affecting respiration rate whilst photosynthesis is not occurring) that may be influencing discolouration (Sharples 1965), although a later study found no such correlation (Jenni 2005). Furthermore, whether lettuce is sensitive to cumulative high temperature exposure or single instances of high temperature exposure has not yet been established.

#### Light

Whilst sunlight levels may impact on the temperature experienced by growing plants, variations in light intensity (Woltering and Witkowska 2016) and wavelength (Koukounaras et al. 2016) during crop growth may have a more direct effect on shelf life. A number of studies have shown that blue and ultraviolet (UV) wavelengths (particularly in the UV-B region) result in increased levels of phenolic compounds (Du et al. 2014; Ouzounis et al. 2015; Koukounaras et al. 2016) and increased PAL activity (Lee et al. 2014), although other wavelengths may influence the levels of other compounds e.g. green wavelengths, resulting in increased carotene and anthocyanin production (Samuolienė et al. 2013). Furthermore the effect of UV exposure may also partially explain the variation in phenolic compound levels through the head of mature lettuce observed by Viacava et al. (2014), since the outer leaves will be exposed for longer and to greater UV intensities than the inner leaves. Plants showing UV-induced increases in phenolic levels do not always show concomitant increases in post-harvest discolouration (Koukounaras et al. 2016), suggesting that light exposure is one of multiple factors at play in inducing subsequent discolouration.

#### Bacterial colonization

Although discolouration is generally considered a physiological disorder in lettuce, there is also some evidence of an association between lettuce discolouration and infection by the bacterium *Pseudomonas marginalis* (Marlett and Stewart 1956; Hall et al. 1971). This bacterial species was associated with browning of inoculated heads stored at room temperature (15–22 °C) and pinking when the heads were stored at 2–8 °C (Hall et al. 1971). *Pseudomonas marginalis* is a widespread organism and the authors suggested that it enters lettuce through breaks in tissue and crushed leaves or cut stems. It is unclear, however, if the observed discolourations are a result of the induction of the wound response pathways in response to tissue damage (for example frost damage), that also allowed the bacteria access to the plant tissue. It is also possible that the presence of the plant pathogenic bacteria exacerbated the wound response and it may be that many different organisms other than just *P. marginalis* could produce such an effect. Non-plant-pathogenic bacteria inducing wound response pathways in such a manner, have been reported in other plant species (Jung et al. 2003; Takemoto et al. 2003).

## Influence of genetics

Although much of the literature concerning discolouration focuses on agronomic characteristics, the vast majority, if not all of these influences have their bases in the genetics of the plant. Cultivated lettuce is a diploid species that naturally inbreeds to a high level. Selection over many decades, even centuries, has resulted in six edible forms of lettuce in the species *L. sativa:* crisphead (Batavia and iceberg types), butterhead, romaine, leaf, latin and stem (Ryder 1999). Traditional selective breeding has resulted in the huge range of inter- and intra- specific variation resulting in the production of hundreds of uniform varieties (Watts 1980; Ryder 1999). In more modern breeding strategies, the wild species *L. serriola* L., which is thought to be the progenitor of *L. sativa* (Kesseli et al. 1994; van de Wiel et al. 1998), has been used extensively as a source of beneficial alleles in commercial lettuce breeding, for example against the oomycete pathogen *Bremia lactucae* (downy mildew) (Lebeda and Pink 1998).

To date, there have been few studies of varietal effects on lettuce postharvest quality, however differences, particularly in respect of both the appearance and rate of pinking, have been noted (Ryder and McCreight 1996; Atkinson et al. 2013a). Although Wurr et al. (2003) found no relationship between tissue strength or stiffness and discolouration, physical leaf properties such as break-strength, plasticity, elasticity and cell area have been correlated with shelf-life in a cross between *L. sativa* and *L. serriola*, suggesting lettuce types with small cells and strong cell walls have better shelf-life (Zhang et al. 2007). This observation supports the suggestion that cellular integrity may be important in reducing discolouration and Wagstaff et al. (2010) noted that shelf-life increased by up to 66% with a concomitant reduction in membrane permeability in transgenic lettuce plants expressing reduced levels of the enzyme xyloglucan endotransglucosylase/hydrolase, which is involved in regulating cell wall rigidity. Variation in levels of PAL activity, phenolic and flavonoid compounds and have also been shown between lettuce cultivars (DuPont et al. 2000; Liu et al. 2007). López-Galvez et al. (1996) noted that the variation in PAL activity was also associated with different head morphologies; activity being lower in iceberg types than romaine types with butterhead types showing higher levels. Cultivar-specific differences in phenolic profiles have also been observed in lettuce responses to light wavelength (Ouzounis et al. 2015; Koukounaras et al. 2016).

Cultivar performance/phenotype can vary across environments due to different responses to numerous biotic, climatic and soil factors (Dixon et al. 1991). If genes are environmentally sensitive, this phenotypic plasticity is expressed at a genetic level in response to environmental variation i.e., a genotype × environment (G × E) interaction (Maloof 2003; Juenger et al. 2005). G × E can limit deployment of potential crop improvements since commercially successful cultivars or varieties must be robust under many if not all growing environments. In development, it becomes necessary to separate genetic effects from environmental ones (Yang and Zhu 2005; Collard and Mackill 2008). Since environmental factors can often be quantified, QTL mapping offers the potential both to provide useful insight into the impact of particular environmental factors, and to identify genetic regions that respond to those factors. Plant breeders could also potentially exploit identified plant genes that respond to the environmental element in G × E interactions either by developing varieties specifically suited to particular environments or by incorporating these genes in additional breeding programmes for environmental traits.

### Breeding strategies

Crop production strategies and post-harvest treatments aimed at prolonging shelf-life introduce an associated cost to production. A more cost-effective method of reducing discolouration and increasing shelf life may be the production of varieties that show a reduced propensity to discolour or a slower development of discolouration. This approach would reduce production costs albeit with an increased cost to breeders, which may be passed on to producers and customers. The additional breeding costs however would be a single increase for each improvement, whilst cost reductions from reduced waste and improved shelf-life would be on-going.

Traditional breeding programmes based on phenotype selection however, are costly and time consuming, generally requiring 6–8 generations for each trait introduced. In addition they are generally more efficient at introducing traits which are expressed as easily scored qualitative variation than those which show quantitative phenotypes. Marker-assisted selection (MAS) is an indirect selection process where a trait of interest is selected for based on a molecular marker which is co-inherited with the target trait (Ribaut and Hoisington 1998; Reynolds et al. 2001; Rosyara 2006). Because MAS is based on genotypic not phenotypic selection, it is also generally independent of environment, obviating the need for multiple site breeding trials, although for some traits, such as discolouration, where there is environmental influence, the initial screens to identify linked markers may need to conducted in several environments or in environments which impact other traits under selection in the same programme. Although this represents an increased cost compared to a single screening, it is incurred only once in determining the markers to be used in the screening process. In addition, MAS can be applied to existing cultivar development programmes to screen for additional traits as they are identified, rather than necessitating an entire new programme for each trait (Collard and Mackill 2008) thereby potentially reducing breeding costs. This process relies on markers tightly linked to the gene or genetic region of interest. Complex traits are likely to be influenced by a large number of genes (Kearsey and Farquhar 1998) meaning that quantitative trait loci (QTL) mapping is the most effective way of identifying the genetic regions associated with variation in these traits. The highly complex nature of the biochemistry underlying post-harvest discolouration in lettuce suggests that MAS breeding programmes based on QTL mapping of discolouration phenotypes are the most likely routes to the successful development of varieties with reduced discolouration traits.

The precision of QTL mapping depends on the genetic variation covered by a population, the size of the mapping population and number and density of marker loci (Abdurakhmonov and Abdukarimov 2008). Consequently, high density genetic linkage maps are now becoming essential tools in the breeding process (Langridge et al. 2001). Although lettuce is not considered an ideal model crop species, most cultivars are highly inbred and reveal extensive genetic homozygosity, allowing for genetic tools to be developed (Michelmore et al. 1994). There have been several iterations of lettuce linkage maps developed from intra-specific crosses in *L. sativa* (Landry et al. 1987; Kesseli et al. 1994; Waycott et al. 1999; Hayashi et al. 2008; Atkinson et al. 2013b; Jenni et al. 2013) and from inter-specific crosses between *L. sativa* and wild relatives *L. serriola* (Johnson et al. 2000; Syed et al. 2006; McHale et al. 2009) and *L. saligna* (Jeuken et al. 2001). More recently these have been developed into an ultra-high density consensus map (Truco et al. 2007, 2013; Stoffel et al. 2012) and an annotated genome sequence assembly of lettuce (Reyes-Chin-Wo et al. 2017) is available at the Lettuce Genome Resource (https://lgr.genomecenter.ucdavis.edu).

This means that it is now possible to begin to unravel the genetics of lettuce discolouration and in 2015, Rijk Zwaan, a major vegetable breeding company, released a range of lettuce cultivars possessing the “Knox” trait, which delayed post-harvest discolouration by up to two days (https://www.rijkzwaan.co.uk/solutions/knox). However, the underlying basis of this interesting and potentially beneficial phenotype remains unclear.

The most obvious targets for breeding programmes would appear to be genes in the two major pathways involved in the development of discolouration, the phenylpropanoid and PPO pathways. A recent metabolomic study of two cultivars of iceberg lettuce showed that phenolic compounds generally increased during storage whilst other metabolites in other groups potentially involved as precursors to the development of coloured compounds, such as amino acids, sesquiterpene lactones, fatty acids and phospholipids decreased in concentration (Garcia et al. 2016). PAL is probably the most studied enzyme in these two pathways. PAL however, acts at the beginning of a biochemical pathway that has numerous endpoints including those involved with response to pathogen and pest attack, consequently varieties with reduced PAL activity may have multiple unintended phenotypes for important traits. The other target, PPO, is more promising: A candidate gene for this enzyme has been associated with a QTL for browning in potato (Weirj et al. 2007) and the reactions catalysed by PPO are more clearly defined, however simply breeding for reduced PPO activity may still have unintended consequences. PPO activity has been associated with resistance to plant pathogens, potentially via antimicrobial properties of the resultant quinones (Richter et al. 2012). It has also been suggested that regulation of polyphenol biosynthesis could be an effective method of discolouration control (Hisaminato et al. 2001). The resulting pigment from the activity of PPO has been reported to be dependent on the structure of the starting phenolic substrate (Toivonen and Brummell 2008) suggesting that enzymes responsible for the production of specific phenolic compounds further down the phenylpropanoid pathway from PAL may be more important than PAL itself. Other potential targets may include the enzymes involved in the metabolism of cysteine or ascorbic acid, two inhibitors of PPO activity, or those concerned with chlorophyll breakdown. In addition to enzymes, phenotypic traits with links to discolouration, such as drought tolerance (enabling plants to maintain yield even under mild water-deficit stress), or response to light, should be evaluated, especially where these characteristics are already part of existing breeding programs (e.g. in response to climate change). Even manipulations such as these are not without consequence however, and any such changes may impact on organoleptic characteristics such as flavour and odour or may have other inadvertent effects on plant biochemistry.

Although traits can be included in breeding programmes relatively easily, effective deployment of the trait without introducing concomitant undesirable characteristics and in a form that behaves robustly in multiple environments requires a detailed understanding of the underlying biochemistry and genetics. The current understanding of lettuce biochemistry is not sufficient to enable potential target genes to be identified based solely on biochemical pathways and furthermore, the biochemistry of discolouration is sufficiently complex that the overall effect of variation in a specific enzyme activity, both on discolouration and other plant processes, would be difficult if not impossible to predict. A combinational approach involving mapping phenotypic (discolouration), biochemical and genetic (transcriptional) variation using high density linkage maps is likely to prove more effective in both determining potential targets and identifying individuals with variations which could be exploited in breeding programmes. The web of plant biochemical activity is heavily interconnected and other considerations such as processing requirements and important consumer attributes such as taste and texture (the genetics and biochemistry of which are not completely understood, but which may be influenced by variations in various biochemical pathways) also need to be taken into consideration.

## Summary

The biochemistry of oxidative discolouration is extremely complex involving two major biochemical pathways; PAL and PPO, that provide key direct or indirect targets for potential breeding programs. Our understanding of the biochemistry of the processes involved, however, remains incomplete. In addition, environmental and agronomic factors including light, temperature, water, nutrients and physical damage also have the potential to impact on the development and extent of discolouration.

The development of cultivars which are less prone to, or slower to develop, discolouration as a phenotypic trait is potentially a cost-effective method of reducing wastage. Breeding programs targeting environmental response traits associated with discolouration could also be useful (whether this is in the form of tolerance for factors which cannot be controlled or improved responses to agronomic inputs), however the precise nature of the interactions between the environmental triggers and the discolouration biochemistry still needs to be determined in order for this to be applied in a systematic way.

Plant biochemistry and/or responses to environmental triggers impact on many other traits, not just oxidative discolouration. The chances of inadvertent detrimental outcomes of breeding programs remain considerable. Even with a significantly improved understanding of the biochemistry and interconnectivity of all the pathways involved, it is possible, even likely, that no single solution can be found to discolouration. The extension of shelf life through reduction of discolouration potential and/or delay of onset of the discolouration may be an incremental process, achieving small gains by subtle changes to the biochemistry of the plant whilst balancing those gains against trade-offs in other traits.

In order to maximize the chances of success, multiple breeding approaches need to be taken in parallel. Phenotypic evaluations of cultivars and other accessions with reduced propensity for discolouration need to be undertaken to provide additional breeding material, alongside continued identification of quantitative traits associated with the development of symptoms via the underlying biochemistry, molecular biology and genetics. Furthermore, these approaches need to be investigated in different growing conditions to elucidate the environmental components of phenotype development and all of the above must be balanced against the other requirements of the commercial sector (e.g. taste and texture). Advances in each of these areas will enhance understanding of the processes of oxidative discoloration and eventually improve post-harvest quality of leafy crops.
